# Are skin disorders related to work strain in hospital workers? A cross-sectional study

**DOI:** 10.1186/1471-2458-11-600

**Published:** 2011-07-28

**Authors:** Nicola Magnavita, Marko Elovainio, Tarja Heponiemi, Anna M Magnavita, Antonio Bergamaschi

**Affiliations:** 1 Institute of Occupational Medicine, Universita Cattolica del Sacro Cuore, Roma, Italy; 2 National Institute for Health and Welfare, Helsinki, Finland; 3 University College London, London, UK; 4 Dermatology Department, John Paul II Hospital, Lamezia Terme, Italy

**Keywords:** hospital workers, skin disorders, occupational stress factors, psychological problems, anxiety, depression, demand, control, social support, strain, isostrain

## Abstract

**Background:**

To evaluate whether occupational stress factors (high demands, low control, low social support, strain, and iso-strain) are associated with skin disorders in hospital workers and whether psychological problems, such as anxiety and depression, act as potential mechanisms through which occupational stress factors are associated with skin disorders.

**Methods:**

1,744 hospital workers were invited to answer a questionnaire concerning the occurrence of skin disorders and psychosocial factors at work. The abbreviated Italian version of the Demand/Control model (Karasek) was used to assess perceived work strain, while the Goldberg scales were used to assess anxiety and depression. Analyses were adjusted for age, gender, occupation, latex glove use and history of atopy.

**Results:**

Of the participants, 25% reported hand dermatitis in the previous 12 months and 35% had been affected by skin disorders in other parts of the body. High job demands (OR = 1.09 CI95% 1.05-1.14), low social support (OR = 0.90, CI95% 0.87-0.93), high strain (OR = 1.54 CI95% 1.20-1.98) and high iso-strain (1.66 CI95% 1.27-2.19) were all associated with a higher prevalence of reported hand skin disorders. Both depression (OR = 2.50 CI95% 1.99-3.14) and anxiety (OR = 2.29 CI95% 1.81-2.89) were associated with higher risk of hand skin disorders. The same pattern was observed for dermatological complaints in other parts of the body. Only a slight reduction in the association between occupational stress variables and skin disorders was observed after including depression and anxiety in the model.

**Conclusions:**

Job stress plays a significant role in triggering skin disorders among hospital workers and psychological problems do not appear to be the mechanism behind this association. Occupational health education and training should focus on reducing job demands and occupational stress in order to prevent skin problems among hospital workers.

## Background

Health care workers (HCWs) often complain of skin disorders, which can significantly interfere with professional performance. Workers' hands are frequently exposed to wet work and irritants due to hand hygiene which is essential in preventing cross-infections. Glove use may cause irritant hand eczema (due to occlusion), contact dermatitis due to rubber accelerators, and latex allergy. Since a considerable percentage of HCWs complain of dermatological problems during periodical medical examinations at the workplace, an accurate analysis should be made of all factors contributing to the induction and exacerbation of work-related skin diseases. The literature provides extensive information on allergic or irritant factors, but little research has been done into other possible causal factors of work-related skin diseases in HCWs.

It has been suggested that psychological states and psychiatric disorders, such as anxiety and depression, are associated with allergic contact dermatitis and atopic dermatitis [1]. Indeed, depressive disorders [2] and anxiety [3] are more common in the population affected by dermatologic disorders. Stressful life events are often involved in the induction or exacerbation of psoriasis, chronic urticaria and other skin conditions [4-6] and occupational stress has been associated with the occurrence of skin symptoms in visual display terminal workers [7] and in musicians [8]. Occupational stress modulates skin response in allergic contact dermatitis [9]. Severe cases of eczema in atopic patients significantly improve after psychological intervention [10].

Evidence suggests that high job strain may also be associated with skin problems, together with other somatic symptoms. For example, previous studies show that high job strain, high demand, or low control are associated with many somatic symptoms including skin problems [11]. Psychological stress seems to worsen several common skin diseases, especially immuno-dermatoses such as psoriasis, atopic dermatitis, eczema and urticaria [12,13] and patients with hand dermatoses are often convinced that stress influences the course of their disease [14].

The aim of this study was to evaluate (1) whether occupational stress factors (demands, low control, low social support, strain, and iso-strain) are associated with skin complaints in HCWs and (2) whether psychological symptoms, such as anxiety and depression, act as potential mechanisms through which these occupational stress factors are associated with skin complaints. (3) A further aim was to evaluate the frequency and type of work-related skin diseases.

## Methods

### Subjects

This cross-sectional survey is part of a longitudinal study of HCWs, involving 1,909 workers employed in 3 hospitals in the Latium Region of Italy. These workers were exposed to occupational risk factors and consequently subjected to compulsory periodical medical examinations.

Before their medical examination In 2005, they were invited to compile a questionnaire concerning the occurrence of skin disorders and psychosocial factors at work. Four subjects refused to fill in the questionnaire, 36 gave incomplete responses, 125 failed to perform their periodical medical examination. 1,744 hospital workers (767 male, 977 female workers; age 44.9 ± 8.9 years) were studied (91.3% response rate). The study population included nurses (44%), physicians (17%), clerks (11%), ancillary nurses (9%), technicians (6%), head nurses (4%), blue collar workers (3%), biologists (2%), and other occupations, including psychologists, social assistants, and many others (3%).

Workers were examined soon after completion of the questionnaire. The first inspection of the skin was performed by an occupational physician. Workers showing objective changes were then examined by a dermatologist. Both physicians were blinded to the results of the questionnaire. Selected cases of suspected allergy underwent skin prick tests, patch tests and IgE measurement.

### Measurements

Both *use of hand gloves *and *history of atopy *were self reported in the questionnaire. Workers were classified as "glove users" if they reported frequent (daily) use of latex, nitrile or vinyl gloves.

The general risk of developing atopy was assessed by the four-items scale contained in the IAQ-MM40 Indoor Air Questionnaire [15,16] (alpha reliability = .56). Workers reporting a history of hay fever, seasonal rhinitis and conjunctivitis, asthma, atopic dermatitis, or family history of atopic diseases were classified as "probably atopic".

Perceived work strain was assessed by the abbreviated Italian version [17] of the Demand/Control/Support model [18]. *The psychological demand scale *had five items (alpha reliability was 0.76), the *control scale *had six items (α = 0.67) and *the support scale *had six items (α = 0.87). A participant was defined as having *high job strain *if the participant scored high on the job demands and low on the job control (defined as above the median score on the respective scales). Participants who reported low levels of social support (median split) together with job strain (high job demands and low job control) were defined as having *high iso-strain*.

*Anxiety and depression *were assessed using the Italian version [19] of the Goldberg scales [20]. This short interview, designed to be used by non-psychiatrists, is composed of two scales of 9 binary items; a score of one is recorded against each question answered in the affirmative. People with anxiety scores of five, or depression scores of two have a 50% chance of having a clinically important disturbance; above these scores the probability rises sharply [21]. Consequently, workers who scored five or more were classified as "anxious", while workers who scored two or more were classified as "depressed". The internal consistency reliability coefficient (Cronbach's alpha) value was 0.82 for the anxiety scale, and 0.78 for the depression scale.

### Statistical analyses

The association of work-related and individual variables with the 12-month prevalence of skin complaints (divided into hand dermatitis, dermatitis in other parts of the body, and both), and with the objective presence of skin disorders (hand dermatitis, and disorders in other parts of the body) was studied using two-step logistic regression analyses. Model I analyzed the univariate effects and Model II was adjusted for age, gender, occupation, glove use, and probable atopy. The potential mediating effect of depression and anxiety was tested following the recommendations of Baron & Kenny [22]. There is evidence to support the hypothesized mediating effects if the following conditions are met: firstly, occupational stress factors (demands, low control, low social support, strain, and iso-strain) are related to skin disorders; secondly, mediating variables (depression and anxiety) are associated with occupational stress factors and with skin disorders; thirdly, inclusion of mediating variables in the analysis reduces the association between occupational stress factors and skin disorders. Analyses to test the third condition were adjusted for age, gender, occupation, glove use, and probable atopy. All the statistical analyses were performed with PAWS/SPSS package (release 17.0).

## Results

The sample characteristics are presented in Table 1.

**Table 1:** Study characteristics

	N	%	Mean (SD)
Age (years)			44.9 (8.9)
Job category			
Physicians	304	17	
Nurses	767	44	
Ancillary	150	9	
Technicians	97	6	
Clerks	191	11	
Head nurse	78	4	
Blue collars	49	3	
Biologists	37	2	
Others	71	3	
History of atopy			
Yes	877	50	
No	867	50	
Wearing Gloves			
Yes	1246	71	
No	498	29	
Job demands			2.8 (0.6)
Job control			2.7 (0.5)
Social support			3.4 (0.5)
Job strain			
High	386	22	
Low	1346	78	
Iso-strain			
High	289	17	
Low	1455	83	
Depression (cases)	483	28	
Anxiety (cases)	462	27	

436 workers (25.0%) reported the onset of work-related skin disorders on fingers or the palm of the hands in the 12-month period prior to examination. 604 HCWs (34.6%) complained of skin disorders on wrist, elbow, knee, face, or other parts of the body. The two groups partially overlap: 730 workers reported some skin disorder, 310 (17,4%) reported skin changes both on the hands and other parts of the body.

According to the workers, skin disorders were related to the use of gloves (397 cases, 22.8%), wet work and hand-washing (365, 20.9%), use of detergents and disinfectants (171, 9.8%), and other causes (278, 15.9%). Most of the complaints were mild or transient, and no longer evident at medical examination. Changes in the skin of the hands were observed by the occupational health physician in 138 HCWs (7.9% of the whole population, 31.6% of those who reported hand skin complaints), and in other parts of the body in 100 cases (5.7% of the sample, 13% of positive answers at the questionnaire). 31 workers manifested both hand and body changes.

In 95 cases (5.4% of the population), dermatological examination of the hands revealed irritative changes, ranging from mild dryness or transient redness to more persistent changes in hand skin. An allergic origin was suspected in 43 cases; in 21 of these, allergen testing confirmed atopic dermatitis (prevalence rate 1.2%), while latex allergy was observed in 5 cases (0.3%).

Low-protein powder-free NRL gloves or NRL latex-free gloves were prescribed for 98 HCWs. At the subsequent medical examination, one year later, 95 of these workers (69%) showed a marked improvement or healing of skin lesions. Two workers with chronic eczematous lesions, non-sensitized to NRL, showed little or no improvement. Owing to the occurrence of bronchial hyper-responsiveness, one NRL-sensitized nurse had to be removed from clinical tasks.

Dermatological disorders of other parts of the body included psoriasis vulgaris (39 cases, 2.2% of the population), urticaria, acne, pityriasis, onychomicosis, and other less frequent dermatoses.

Table 2 shows the associations between occupational stress factors and skin disorders. Higher job demands and lower social support, high strain and high iso-strain were all associated with a higher prevalence of skin complaints in all categories. The strongest association was detected between iso-strain and skin disorders (ORs from 1. 66 to 2.81). Job control was not associated with complaints in the 12-month period before examination; there was, however, a significant inverse relationship with disorders observed during medical examination. Thus, the first condition of mediation was met regarding all other variables than job control (stress variables were associated with skin disorders). Both depression and anxiety were also associated with higher risk of skin complaints. No change occurred in these associations after adjustments for age, gender, occupation, use of gloves and probable atopy.

**Table 2:** Odds ratios (OR) and 95% CIs for the associations of occupational stress factors and psychological problems with skin complaints and skin disorder categories

	**Skin complaints -12 month prevalence**			**Skin disorders- point prevalence**	
	
**Individual** **and job characteristics**	**Hand complaints** **(436 cases)**	**Complaints in other body parts** **(604 cases)**	**Any complaint** **(730 cases)**	**Hand dermatitis** **(138 cases)**	**Dermatitis in other parts** **(100 cases)**
	
	**OR (95% CI)**	**OR (95% CI)**	**OR (95% CI)**	**OR (95% CI)**	**OR (95% CI)**
	
	Model I^a^	Model I	Model I	Model I	Model I
Job control	0.97 (0.94 - 1.00)	0.96 (0.93 - 0.98)	0.97 (0.94 - 1.00)	0.93 (0.89-0.98)	0.92 (0.87-0.98)
Job demands	1.09 (1.05 - 1.14)	1.12 (1.08 - 1.16)	1.12 (1.08 - 1.16)	1.15 (1.07-1.23)	1.14 (1.06-1.23)
Social support	0.90 (0.87 - 0.93)	0.92 (0.88 - 0.94)	0.91 (0.88 - 0.94)	0.86 (0.83-0.90)	0.89 (0.84-0.94)
High strain	1.54 (1.20 - 1.98)	1.76 (1.40 -2.22)	1.62 (1.29 -2.03)	1.98 (1.37.2.87)	2.16 (1.42-3.30)
High iso-strain	1.66 (1.27 - 2.19)	1.91 (1.48 - 2.46)	1.77 (1.37 - 2.28)	2.22 (1.50-3.29)	2.81 (1.82-4.33)
Depression	2.50 (1.99 - 3.14)	2.13 (1.72 - 2.64)	2.21 (1.78 - 2.73)	1.27 (1.18- 1.36)	1.29 (1.19-1.40)
Anxiety	2.29 (1.81 - 2.89)	2.02 (1.63 - 2.52)	2.09 (1.69 - 2.60)	1.24 (1.16-1.32)	1.24 (1.16-1.33)
	Model II^b^	Model II	Model II	Model II	Model II
Job control	0.97 (0.94 - 1.01)	0.95 (0.92 - 0.99)	0.97 (0.94 - 1.00)	0.93 (0.88-0.98)	0.94 (0.88-0.99)
Job demands	1.08 (1.04 - 1.13)	1.11 (1.07 - 1.16)	1.11 (1.07 - 1.16)	1.13 (1.06-1.22)	1.14 (1.05-1.24)
Social support	0.90 (0.87 - 0.94)	0.91 (0.88 - 0.94)	0.91 (0.88 - 0.94)	0.87 (0.82-0.91)	0.90 (0.85-0.95)
High strain	1.50 (1.16 -2.95)	1.79 (1.40 -2.29)	1.64 (1.29 -2.09)	1.91 (1.29-2.91)	2.03 (1.31-3.15)
High iso-strain	1.56 (1.17-2.07)	1.89 (1.45 - 2.48)	1.74 (1.33 - 2.28)	2.07 (1.37-3.11)	2.62 (1.67-4.11)
Depression	1.90 (1.72 - 2.80)	1.93 (1.53 - 2.43)	1.98 (1.57 - 2.49)	2.57 (1.77-3.73)	2.61 (1.70-4.03)
Anxiety	2.04 (1.59 - 2.61)	1.90 (1.51 - 2.41)	1.95 (1.55 - 2.46)	2.54 (1.75-3.68)	2.65 (1.72-4.09)

^a ^Model I: Univariate associations; ^b ^Model II: adjusted for age, gender, occupation, glove use, and probable atopy.

Depression was associated with job control (OR 0.92, 95% CI 0.90 - 0.95), job demands (OR 1.19, 95% CI 1.15 -1.25) and social support (OR 0.86, 95% CI 0.84 - 0.89). Similarly depression was associated with high strain (OR 2.16, 95% CI 1.70 -2.75) and iso-strain (OR 2.64, 95% CI 2.04 - 3.43). Anxiety was associated with job control (OR 0.90, 95% CI 0.87 - 0.93), job demands (OR 1.20, 95% CI 1.15 - 1.25), social support (OR 0.83, 95% CI 0.81 - 0.87), high strain (OR 2.41, 95% CI 1.89 - 3.06) and high iso-strain (OR 2.87, 95% CI 2.21 - 3.73). Thus, the second condition for mediation was met.

The hierarchical logistic regression analyses used to test the third condition for the potential mediating effects of depression and anxiety in the association between occupational stress factors and skin disorders are presented in Table 3. Job control was excluded since it did not meet the criteria for the first condition of mediation. Since only a slight reduction in the ORs was observed after adding the depression and anxiety variables to the model, these psychological states do not appear to account for all the association between job stress and skin disorders.

**Table 3:** Odds ratios (OR) and 95% CIs for the associations of occupational stress factors with skin disorder categories adjusted for age, gender, occupation, glove use, and probable atopy

	**Skin complaints -12 month prevalence**			**Skin disorders- point prevalence**	
	
**Job characteristics**	**Hand complaints** **(436 cases)**	**Complaints in other parts** **(604 cases)**	**Any complaint** **(730 cases)**	**Hand dermatitis** **(138 cases)**	**Dermatitis in other parts** **(100 cases)**
	
	**OR (95% CI)**	**OR (95% CI)**	**OR (95% CI)**		
	
	Model I^a^	Model I	Model I	Model I	Model I
Job demands	1.06 (1.01 - 1.10)	1.10 (1.05 - 1.14)	1.09 (1.05 - 1.14)	1.09 (1.02-1.18)	1.11 (1.02-1.20)
Social support	0.92 (0.89 - 0.95)	0.93 (0.89 - 0.96)	0.92 (0.89 - 0.95)	0.88 (0.84-0.93)	0.91 (0.86-0.97)
High strain	1.36 (1.04 -1.78)	1.67 (1.30 -2.14)	1.53 (1.19 -1.95)	1.65 (1.11-2.45)	1.78 (1.14-2.79)
High iso-strain	1.36 (1.01 - 1.83)	1.72 (1.31 - 2.26)	1.58 (1.20 - 2.07)	1.73 (1.14-2.63)	2.25 (1.42-3.57)
	Model II^b^	Model II	Model II	Model II	Model II
Job demands	1.06 (1.01 - 1.11)	1.10 (1.05 - 1.14)	1.09 (1.05 - 1.14)	1.10 (1.02-1.18)	1.10 (1.02-1.20)
Social support	0.92 (0.89 - 0.95)	0.93 (0.90 - 0.96)	0.92 (0.89 - 0.95)	0.88 (0.84-0.93)	0.92 (0.87-0.97)
High strain	1.37 (1.04 -1.78)	1.66 (1.29 -2.20)	1.51 (1.18 -1.93)	1.65 (1.11-2.45)	1.76 (1.13-2.76)
High iso-strain	1.38 (1.03-1.85)	1.72 (1.31 - 2.26)	1.57 (1.90 - 2.07)	1.75 (1.15-2.65)	2.23 (1.41-3.53)
	Model III^c^	Model III	Model III	Model III	Model III
Job demands	1.05 (1.00 - 1.10)	1.09 (1.05 - 1.14)	1.09 (1.05 - 1.13)	1.08 (1.01-1.17)	1.09 (1.01-1.19)
Social support	0.92 (0.89 - 0.96)	0.93 (0.90 - 0.96)	0.92 (0.89 - 0.96)	0.89 (0.84-0.94)	0.92 (0.87-0.98)
High strain	1.32 (1.01 - 1.73)	1.63 (1.27 - 2.09)	1.49 (1.16 - 1.90)	1.57 (1.06-2.35)	1.70 (1.08-2.66)
High iso-strain	1.32 (0.98 - 1.77)	1.67 (1.27 - 2.20)	1.53 (1.16 - 2.02)	1.64 (1.08-2.51)	2.13 (1.34-3.39)

^a^Model I was additionally adjusted for depression; ^b^Model II was additionally adjusted for anxiety; ^c^Model III was additionally adjusted for both depression and anxiety.

## Discussion

Our results suggest that skin complaints in HCWs are strongly related to work stress as defined by the widely studied JDC-model. High demands, high strain, and the combination of strain with isolation (iso-strain) increased the reporting of skin disorders in the year prior to medical examination, whereas social support exerted a protective effect. The observed associations were significant even after adjustment for age, gender, occupation, use of latex gloves, and history of atopy. Psychological symptoms, such as depression and anxiety, did not act as mechanisms behind these associations.

Our findings also demonstrate the importance of emotional states, such as depression and anxiety, in the occurrence of skin disorders. Our results indicate that the prevalence of skin disorders is more than double in depressed workers. A similar pattern was found between anxiety and skin disorders. Our findings are in line with previous studies thereby suggesting an association between psychiatric disorders and chronic skin diseases. For example, high levels of depression and anxiety have been observed in patients with skin diseases such as psoriasis vulgaris [23], atopic dermatitis [3], lichen planus [24] and Behcet's disease [25].

However, in our analysis, neither depression nor anxiety accounted for the association between occupational stress variables and skin disorders that remained significant even after adjustment for depression and anxiety. It can be argued that job stress is associated with skin disorders either directly or through other mechanisms (lack of time to wash hands properly, inability to make use of breaks, using the same gloves for too long). Consequently, organizational and relational job-related factors could play a significant role in triggering or exacerbating skin diseases. Our findings imply that a high level of stress or inappropriate handling of job demands, conditions of stress and emotions during conflict and in decision-making situations can have a detrimental effect on the onset or the course of skin disorders.

The strength of our study is that even though several studies on the correlation between stress and skin disorders have been published in recent years, to our knowledge, only a few (if any), have analyzed the association between job strain and skin disorders. However, some limitations should be taken into account when interpreting our results. Firstly, the retrospective character of reporting may be influenced by recall bias. Secondly, we had to use a very simple questionnaire, comprising only a few questions, since it had to be filled in during the brief waiting interval before each examination at the workplace. The generic "skin disorder" answer is very comprehensive and fails to allow a clinical classification of diseases. However, workers with objective skin changes underwent dermatological and allergological studies and were given a diagnosis. Thirdly, a cross-sectional survey cannot argue on causal inferences. However, objective examination, followed by dermatological and allergological evaluation and many years of medical surveillance, confirmed that there was no objective evidence for many complaints and that most workers had only mild disease. This undoubtedly supports the hypothesis that work stress causes complaints. However, the results of the present study need to be confirmed in the future with a prospective study design.

From a general point of view, self-reports can lead to concern about their validity. Personality traits, such as a disposition towards neuroticism and negative moods, may affect the perception of occupational stress factors and cause an overestimation of skin disorders. However, we tested the effects of depression and anxiety, the psychological states that are the commonest correlates of negative mood and neuroticism, and they did not account for the association between stress variables and skin disorders.

There are also some other potential mediators that were not controlled in this study. They include those that may increase sensory innervations or the production of pruritogenic agents, that perpetuate neurogenic inflammation and lower the itch threshold [26]. Thus, future studies should use more objective measurements and also control exposure to the above-mentioned factors more fully.

## Conclusions

In conclusion, our study demonstrates that so-called "glove-dermatitis", so common in HCWs, is not merely an irritative or allergic disease, but a more complex condition that involves individual and organizational factors. The prevention of hand dermatitis in hospital workers should be based on a multi-level approach. The same statement is true for other skin disorders in health care workers. Occupational health education and occupational training programmes may be necessary to improve the knowledge and ability of HCWs to cope with job demands and reduce occupational stress.

## Competing interests

The authors declare that they have no competing interests.

## Authors' contributions

NM was the occupational health physician who had the study idea, collected the data, and drafted the manuscript; ME participated in the design and coordination of the study; TH made a substantial contribution to statistical analysis and interpretation of the data, and participated in the study design; AMM was the dermatologist who revised the data. Antonio Bergamaschi, Director of the Department of Occupational Medicine, participated in drafting the manuscript. All authors have given final approval of the version to be published.

## Funding

None.

## Pre-publication history

The pre-publication history for this paper can be accessed here:

http://www.biomedcentral.com/1471-2458/11/600/prepub
